# Detecting trends in academic research from a citation network using network representation learning

**DOI:** 10.1371/journal.pone.0197260

**Published:** 2018-05-21

**Authors:** Kimitaka Asatani, Junichiro Mori, Masanao Ochi, Ichiro Sakata

**Affiliations:** The Graduate School of Engineering, The University of Tokyo, Tokyo, Japan; Universidad Rey Juan Carlos, SPAIN

## Abstract

Several network features and information retrieval methods have been proposed to elucidate the structure of citation networks and to detect important nodes. However, it is difficult to retrieve information related to trends in an academic field and to detect cutting-edge areas from the citation network. In this paper, we propose a novel framework that detects the trend as the growth direction of a citation network using network representation learning(NRL). We presume that the linear growth of citation network in latent space obtained by NRL is the result of the iterative edge additional process of a citation network. On APS datasets and papers of some domains of the Web of Science, we confirm the existence of trends by observing that an academic field grows in a specific direction linearly in latent space. Next, we calculate each node’s degree of trend-following as an indicator called the intrinsic publication year (IPY). As a result, there is a correlation between the indicator and the number of future citations. Furthermore, a word frequently used in the abstracts of cutting-edge papers (high-IPY paper) is likely to be used often in future publications. These results confirm the validity of the detected trend for predicting citation network growth.

## 1 Introduction

A large part of technological development in our society is based on academic research [1, 2]. Recently, quite a few academic fields that attract much attention, such as nanotechnology and computer science, have changed rapidly [3]. However, to predict the future of science and technology, it is important to comprehend trends in academic fields and to detect cutting-edge areas (the tip of the trend). The method of predicting the number of citations of papers [4–8] has been studied widely. However, most of these methods consider information related to the local centrality of papers. They are incapable of grasping trends of whole academic domains. Some ad-hoc methods which grasp trends by detecting hot clusters have been proposed: detecting clusters in which recently published papers are likely to located [9] or estimating responses to a paper within/outside the community based on text content [10]. However, numerically grasping the trend that comes from network growth still presents a great challenge.

In this paper, we propose a novel framework that detects the trend (growth direction) from the citation network. The growth direction is presumed to be stored in a network structure because the network expands concomitantly with the iterative process of citation. An edge is created only between the new additional node and existing nodes. The iterative process by which a paper that follows the trend is likely to cite papers on the trend, thereby establishes and reinforces the trend. Earlier studies have demonstrated the difficulty of grasping the growth direction in the citation network. For example, using a network visualization method such as a spring model [11], one can observe areas in which recently published papers are gathered [12]. However, these areas are separated in two-dimensional (2D) embedded space. They demonstrate the difficulty in quantifying each paper’s degree of trend-following. Furthermore, the local process of citation network growth [13, 14] and citation network topology [15] are well researched. From a different approach, producing a geometric graph model [16] provides information related to the classification of citations and citation diversity of each paper. However, these methods cannot elucidate the growth direction of the whole network. Presumably, a richer representation of the network structure is necessary to grasp the network growth direction.

Recently, NRL [17–20], which calculates a high-dimensional vector representation of a network, has received much attention. The positions of nodes in high-dimensional latent space include rich information because the high-dimensional vector representation enables us to use the precision tasks of clustering, label estimation, and visualization [21, 22]. In these tasks, the representation vector of each node is transformed into useful information for these tasks using machine-learning methods. We assume that the information related to the growth direction is included in the latent representation of each node. Especially in LINE [17], the proximity function between nodes is almost wholly dependent on the existence of an edge. Consequently, in the iterative node addition process in the citation network, the direction from a new node to existing nodes is affected by the direction from nodes which the node cites existing nodes. During this repetition, we assume that the citation network grows into specific directions in latent space obtained by LINE.

Hence, we propose the framework as follows: We train a regression model that fits each paper’s latent vector obtained by NRL to each paper’s publication time. The model would grab the growth direction of the citation network. Subsequently, we estimate each paper’s intrinsic publication year (IPY), by fitting the regression model to each paper’s latent vector. Therefore, IPY represents the degree of trend-following. Papers that have a high IPY value are inferred to be located on the cutting edge. Verifying the validity of information extracted by the framework, we confirm that IPY is useful for predicting the future citation count and future frequently used words in the domain.

From APS paper citation datasets or papers of some domains of the Web of Science, we confirmed the existence of trends by observing linear growth of the network in latent space. Next, we calculated each paper’s degree of trend-following as indicator IPY and found the correlation between the indicator and the number of future citations. Moreover, words in the abstracts of papers which have high IPY values are likely to be used often in future publications. In other words, papers located in the cutting-edge, the vanguard of the growth direction of the network on latent space, will be cited many times. According to the explanation above, our method retrieves information related to a trend of network growth. The information is valid for predicting growth of the academic field.

## 2 Detecting growth of an academic field in latent space

**Fig 1:**
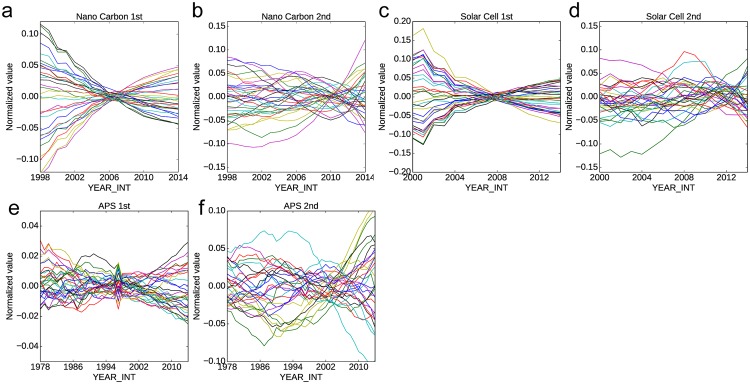
Yearly average of the representation vector of each dimension (Dimensions are randomly sampled). (a,b) Nanocarbon: Result of LINE 1st (a) LINE 2nd(b) (c,d) Solar cells (e,f) APS. The horizontal axis shows the publication year. The vertical axis shows the normalized value of the mean value of the representation vector published in that year.

To confirm that an academic field is growing in a specific direction in each dimension on the representation vectors, we investigated the yearly average of each dimension of a 512-D representation vector calculated using LINE 1st and LINE 2nd. Fig 1 shows the yearly average of the representation vector of each dimension. The yearly average values of each dimension are normalized by means and standard deviations. Fig 1(a) shows the average value of each dimension likely to grow linearly in a specific dimension. This tendency was observed for LINE 2nd (Fig 1(b)) during the last few years. The time span in which the positions of papers change linearly in LINE 1st is longer than that in LINE 2nd in each dataset. The untidy line of the APS dataset (Fig 1(e)) is regarded as resulting from the diversity of the physics academic domain. Overall, we confirmed that each dimension obtained by NRL includes information related to the growth direction. Specifically, the papers are moved to a specific direction linearly in each dimension as time advances. This tendency is observed clearly in the time range of the last 5–10 years of the datasets.

**Fig 2:**
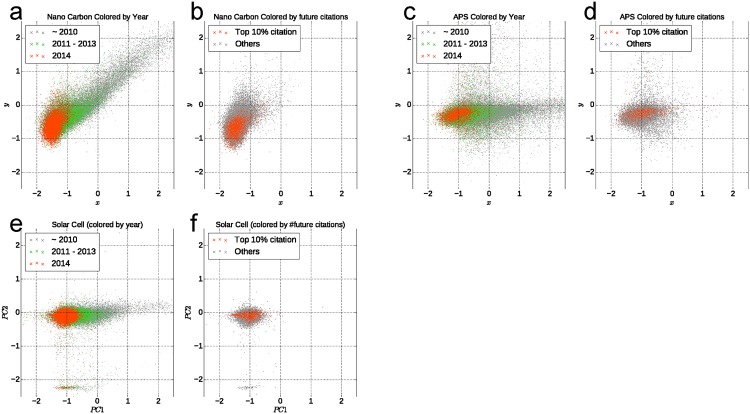
Visualization of papers in academic fields. Papers are colored by year (left panels) and by future citations (right panels): (a) The 2D representation obtained by PCA from the LINE-1st representation of Nanocarbon is shown in this figure. Each dot represents a paper. It is colored by publication year. The group paper grows in specific directions as the year grows. (b) This figure shows only the latest (2014) year’s paper’s 2D representation. The future top 10% cited papers are colored by orange and these papers appear to gather in a specific area. (c,d) Same plot of solar cells and (e,f) APS.

Next, we show representation vectors calculated using LINE 1st and LINE 2nd in 2D space by PCA. To elucidate the direction of growth of the citation network, papers are divided into three groups: those published in 2014 (the newest year in the datasets), those from 2011–2013, and those before 2010. The three groups are presented in different colors in Fig 2(a). As shown in Fig 2(a), papers published in 2012 (red) and 2009–2011 (green) are mapped on the left side for comparison with older papers. These results signify that the growth direction of academic fields can be ascertained using a latent representation of networks. However, the result obtained using LINE 2nd does not signify the growth direction clearly in the primary and secondary axis of PCA.

Additionally, we found that papers that are cited many times in the future are located in a specific area in latent space. In Fig 2(b), 2(d) and 2(f), the top 10% future-cited papers (shown in red points) appear to be located in specific areas. In Fig 2(b), these papers appear to be located on the left side, which is the growth direction compared to all papers. However, these tendencies are not always the same in other datasets.

The results shown above indicate that the NRL method can extract temporal information from the network structure. Highly cited papers appear to be located in specific areas in the nanocarbon dataset. However, this is not observed in other datasets, perhaps because the 2D representation does not include sufficient information to locate the cutting-edge area.

## 3 Predicting future trends: Citation count and keywords

From the results presented above, we found that each dimension of the latent space obtained by LINE 1st or 2nd grows in the specific direction. We propose that the indicator IPY, which represents each paper’s degree of trend-following, has the predictive power for future citations and frequently used words in future publications. Additionally, we show the keywords often used in high-IPY papers that are likely to be important keywords several years later.

### 3.1 Calculation of the IPY indicator

We estimate the growth direction using linear regression analysis with the latent vector as an explanatory variable and with the publication time as a target variable. We choose the parameter cut-off year *C*_*y*_ = 2006(APS) *C*_*y*_ = 2010(Solar cell and Nanocarbon) because the position of a paper in each dimension of the representation vector is likely to grow linearly after 2006(APS) and 2010(Others) (in Fig 2). The parameter selection is discussed in the last part of this subsection. As a regression result, we acquire models that estimate the publication year of each paper with the fitting accuracy of *R*^2^ = 0.2 − 0.4 in each dataset. This result indicates that the position in latent space obtained by NRL includes information related to publication years. After the fitting, we calculate IPY by fitting the model to each paper’s latent representation.

### 3.2 IPY and future citation

**Fig 3:**
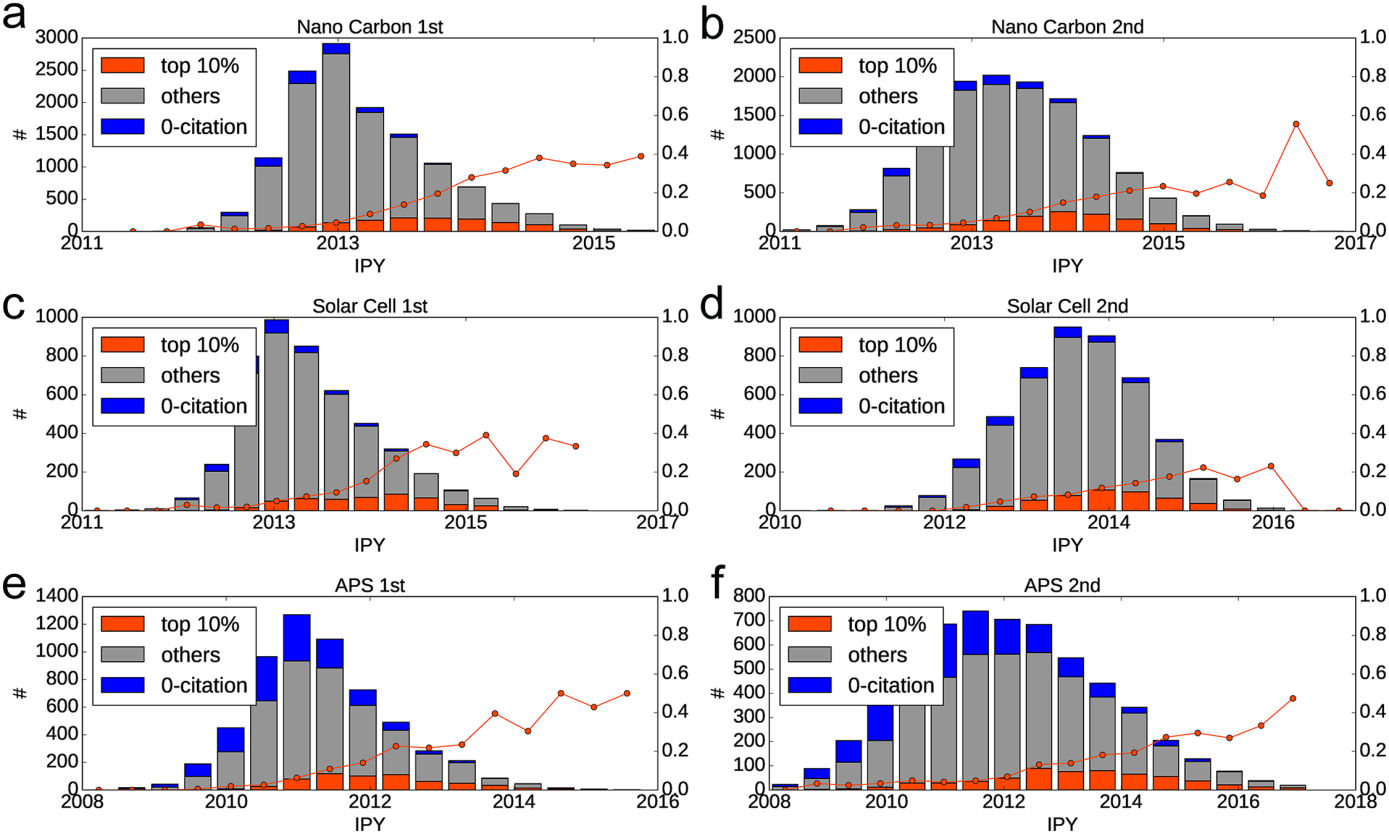
Distribution of IPY in set 0-citation, top 10% future citation, and other papers. The blue line (secondary axis) corresponds to the ratio of future top 10%cited papers in each bin. (a,b) Results of nanocarbon using (a) LINE 1st and (a) LINE 2nd. (c,d) The same plot of Solar cells and (e,f) APS.

First, we demonstrate the relation between IPY and the number of future citations. We analyzed the group of papers published in a short time range (September 2012—December 2012) to demonstrate the citation prediction after soon publishing. We show the distribution of IPY between three groups in Fig 3: 0-citation (no citation over the following three years), top 10% (top 10% number of citations over the following three years), and others. Top-10% citation groups take the highest IPY values: 0-citation groups take the lowest values in LINE 1st results. This tendency is observed for all datasets. This result demonstrates that a high-citation paper is likely to follow the trend. Regarded from another perspective, in the top left panel in Fig 3, more than 30–40% papers having high IPY value (e.g., more than 2014) are future top 10%cited papers. We found this tendency in other datasets. The result obtained using LINE 2nd is not clear compared to LINE 1st. Furthermore, we found that IPY is a good indicator for predicting future citations. Therefore, the papers that follow the trend are cited many times and vice versa.

To compare the indicator with other network features, we predict future highly cited papers from papers that have just been published without the citation of those papers. Target prediction values are the number of citations in October 2017 (Solar cell and Nanocarbon) or December 2016 (APS). To facilitate comparison between our method and relevant baselines, we chose baseline features from previous studies. Basic features that are commonly used in citation prediction were used as features [6, 23, 24], embedding features defined in this paper. In addition to existing features, we defined APYR, which is a naive estimator of the paper’s degree of trend-following: The average publication year of the reference of the paper. The same tendency is observed for other datasets.

The experimentally obtained results of each dataset are given in Table 1. IPY (1st) correlates more strongly to the number of total citations than to other features. This result demonstrates that the features defined in this paper using a latent representation of the network are superior to existing features for predicting the impact of a paper. This result supports the hypothesis that future publications are expected to be located close to a cutting-edge paper at the moment and close to a cutting-edge paper cited by future publications. In all datasets, APYR correlates weakly to the number of future citations, which indicates that IPY can retrieve trend information from the iterative process of trend-making by which trend-following papers cite trend-following papers.

**Table 1:** Correlation between features and log(future citation count + 1).

dataset index	APS	Nanocarbon	Solar Cell
IPY(1st)	**0.419**	**0.398**	0.346
APY(2nd)	0.322	0.276	**0.360**
APYR	0.314	0.261	0.214
PageRank	0.247	0.263	0.296
Degree	0.275	0.292	0.308
Clustering Coefficient	-0.025	0.058	-0.061
Eigenvector centrality	0.082	0.151	-0.002

**Fig 4:**
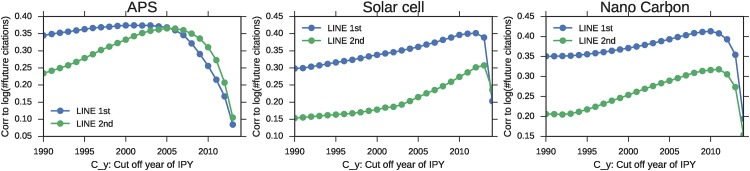
Parameter sensitivity of the cutoff year *C*_*y*_ for the correlation between IPY and future citations. (a) The horizontal axis shows the cutoff year *C*_*y*_. The vertical axis shows the correlation between each paper’s IPY of 2014 papers and the number of 2016 citations. (b,c) This is the same plot of solar cells and APS.

Additionally, we change parameter *C*_*y*_ and measure IPY’s predictability of the future citation. In each of the sub–figures of Fig 4, the horizontal axis shows the cutoff year of IPY (*C*_*y*_). The vertical axis shows the correlation to the log value of the future cited count. From this figure, we can infer that 5–10 years before the target year is the best value of *C*_*y*_ because no dimension of representation vector for the old paper fits linear growth (shown in Fig 2). The best *C*_*y*_ values of LINE 2nd are later than those of LINE 1st in each dataset. This finding coincides with the results showing that each dimension of the vectors calculated using LINE 1st grows linearly after a few years from the beginning of the academic field compared to LINE 2nd.

### 3.3 Predicting word frequency growth in papers

Predicting the rapid increase of words in future papers is important for scientific foresight. We show that words that are likely to be used in cutting-edge research papers will be used in future papers. Predicting the growth of each word’s frequency is a difficult task [25–27]. Using the access log of websites where the paper is presented is another indicator that predicts future words for PubMed papers [28]. Here, we show that keywords often used in high-IPY (cutting edge) papers are likely to be important keywords several years later.

The prediction targets are words appearing in the abstracts of the papers, excluding stop-words. For each word, we calculate the average publication year of the paper that includes the word in the abstract (AIPY). Consequently, words with a high average AIPY are words likely to be used in cutting-edge research papers. We also defined the naive estimator of the novelty of words, APY, which is the average publication year of the paper that includes the word in the abstract. We evaluate the accuracy of the indicator by the correlation between the features and growth ratio of the word frequency and the prediction task of future top-N growth keywords. In each dataset, the growth ratios are the ratio of the keyword frequency in papers published after 2016 to that in papers published in 2014. The prediction task evaluated the number of top-N AIPY/APY keywords among the top-N future growth words.

We narrow down the words that have been used more than *T*_*f*_ > 5, 10, 20 times in 2014 papers and execute the evaluations. The results are presented in Table 2. AIPY is more correlated to the growth of word frequency compared to APY in all datasets. The result is shown on the right side of Table 2. In all datasets, the predicted accuracy of future citation keywords using AIPY is higher than that of APY. The predictability of IPY becomes lower as the threshold of the word’s number of occurrences *T*_*f*_ in the current (2014) papers is set lower. Therefore, it is a difficult task to predict the future word frequency of current less frequently used words. However, the AIPY indicator is better than the naive estimator. Practically speaking, keywords used in the cutting-edge area are expected to be used many times in future reports.

**Table 2:** AIPY and APY’s correlation to the growth of word frequency and results from top-N growth word predictions by changing datasets and *T*_*f*_ = 5, 10, 20.

Dataset	*T*_*f*_	AIPY	APY	top-50 growth words		top-100 growth words	
				AIPY	APY	AIPY	APY
Nanocarbon	5	0.234	0.065	7	7	19	16
	10	0.298	0.062	9	5	14	15
	20	0.330	0.089	7	7	21	14
Solar Cell	5	0.189	0.016	12	9	17	14
	10	0.323	0.058	14	8	27	11
	20	0.365	0.080	11	5	23	13
APS	5	0.085	-0.04	6	3	8	4
	10	0.127	0.002	4	4	10	9
	20	0.226	-0.024	6	4	16	7

## 4 Discussion

As described in this paper, we propose a framework that detects the growth direction of a network, which is considered to indicate a trend. Using the APS dataset as well as data from certain domains of the Web of Science, our framework detects research trends and confirms that a paper located in a cutting edge area (tips of growth direction) is likely to be highly cited in the future. Subsequently, we define the novel indicator IPY, which represents the degree of trend-following of each paper. This method is more accurate than existing centrality features for predicting future citations. This result supports the existence of the Bandwagon effect (i.e. individual adoption of something increases as others do the same thing) in academic fields. Papers which follow a popular trend are cited more often because other scientists follow the same research trend.

In the context of network science, our framework is novel because it retrieves temporal information as the growth direction, which is hidden in citation networks. The temporal information is derived from the iteration processes of edge creation in that edges are created only between a new node and existing nodes. Taking a microscopic view, we can easily ascertain a newer node of each pair of neighbor nodes. However, it was difficult to estimate the degree of freshness of nodes in whole citation network. As described in this paper, we proposed a framework that quantifies the freshness of nodes in a citation network. This framework is applicable to other networks that have similar edge creation processes to a paper citation network, such as a patent citation network and the conversion history of SNS.

We do not claim that papers exploring cutting-edge research area are sophisticated, important, or promising. Certain papers have a considerably strong influence on the academic domain although they do not follow current trends. For example, the first paper on perovskite solar cells [29] published in 2009 was not located in cutting-edge research areas during 2009. The IPY value of the paper which calculated at the publication date is bottom 12% of IPY values of other paper published in the same period. However, later papers that discussed perovskites are located in the cutting-edge areas. This example underscores an important limitation of our method. Future studies must develop methods to detect potentially innovative works by which authors concentrate on ground-breaking research without following the trend.

However, the result obtained using our method can provide foresight in diverse scientific fields. With the rapid growth of academic fields, citation prediction at a paper’s publication date is extremely important policymakers, funding agencies, and most certainly for journal editors who must select high-impact papers. For these reasons, the IPY indicator is expected to provide key insights to many people involved with research and the publication those research findings.

## 5 Methods

**Fig 5:**
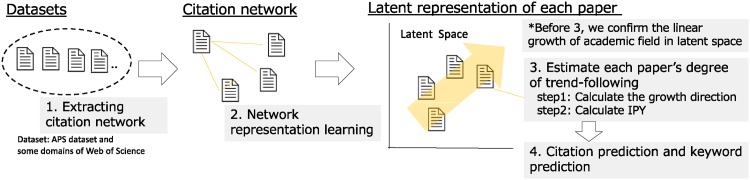
Outline of our citation prediction framework.

The proposed framework detects the trend (growth direction of a network) on latent space mapped from network structure using NRL and quantifies each node’s degree of trend-following as indicator IPY. The framework developed in this study is presented in Fig 5. First, we narrow down data from several datasets to create a sample dataset using a query, and create a citation network. Subsequently, we acquire a distributed representation of each paper using the NRL method. Then we detect the research trend (i.e., the growth direction of an academic field) and estimate the degree of trend-following of each paper based on this direction. Finally, we define the IPY feature and predict the number of future citations of each paper.

### 5.1 Representation learning of a citation network

Direct citation and co-citation networks are important to elucidate the structure of a citation network [30, 31]. To create the representation of both networks, we use LINE [17] because the representative learning methods (LINE 1st and LINE 2nd) in LINE correspond to direct citation networks as well as co-citation networks.

Aside from LINE, several NRL methods have been proposed in the past few years [20]. These methods are roughly divided into matrix factorization methods and methods that define a proximity function between nodes. Some earlier methods [19, 32] are slightly more accurate than others in certain label estimation tasks. However, they are not considered adequate for detecting the growth direction of a time-directed network because these methods do not define local proximities between nodes. A proximity function is defined in both LINE and DeepWalk [18]. Especially in LINE 1st, the proximity function between nodes is almost entirely dependent on the existence of an edge. Considering the iterative process of node addition in citation network, the direction from a new node to existing nodes is affected by the direction from nodes which the node cites to existing nodes. During this repetition, the citation network is presumed to grow in specific directions.

In LINE 1st, the proximity between two nodes is calculated based on the presence of a direct link between the nodes. This corresponds to the analysis of a direct citation network. For citation networks, nodes that are connected by a citation become closer in the embedding result. The closeness of the representation vector of two nodes calculated using LINE 1st represents the probability of a direct link between nodes *i* and *j*. In Eq (1), using representation vectors **v**(*i*) and **v**(*j*), the probability that the nodes are connected is higher if the inner product of **v**(*i*) and **v**(*j*) is large. The representation vectors *v* of each node are estimated as the KL-divergence of *P*_1_(**v**(*i*),**v**(*j*)). Actual data $\hat{P}\left(v\left(i\right),v\left(j\right)\right)=\frac{w_{ij}}{W}\left(W=\sum w_{ij}\right)$ (*w*_*ij*_ = 1 if *i* and *j* are connected; 0 if not connected) takes a small value.

$$\begin{array}{ccc} P_{1}\left(v\left(i\right),v\left(j\right)\right) & = & \frac{1}{1+exp(v{\left(i\right)}^{T}\cdot v(j))} \end{array}$$ (1)

$$\begin{array}{ccc} P_{2}\left(v\left(i\right)|v\left(j\right)\right) & = & \frac{exp(v^{{}^{\prime }}{\left(i\right)}^{T}\cdot v(j))}{\sum_{k=1}^{V}exp(v^{{}^{\prime }}{\left(k\right)}^{T}\cdot v(i))} \end{array}$$ (2)

LINE 2nd is based on the assumption that nodes of high proximity share the same neighbors. Therefore, LINE 2nd corresponds to an analysis of a co-citation network. In Eq (2), *P*_2_(**v***i*|**v***j*) represents the probability of a link from node *j* to node *i*. Each node has representative vectors **v** and **v**′. Of those, **v** represents the way in which outgoing links are created in node *u*; **v**′(*i*) denotes the contents of node *i*. Actually, **v**′ is used only for calculations; **v** is the output of LINE 2nd. For example, if node **v**(*k*) has the same representation vector as **v**(*j*), then the probability of connection to node *i* from node **v**(*j*) and **v**(*k*) takes the same value (*P*_2_(**v**(*i*)|**v**(*j*)) = *P*_2_(**v**(*i*)|**v**(*k*))).

The LINE parameters are the number of dimensions of representative space *N* = 512, the number of iterations of Asynchronous Stochastic Gradient Descent *S* = 10000, and the ratio of negative sampling *N* = 8.

### 5.2 Indicator of the degree of trend-following

We defined the intrinsic publication year (IPY) feature, which represents the degree of trend-following of the paper. IPY is calculated as the year scale (e.g. 2016.3). A larger value of IPY indicates that the paper follows the current trend and vice versa. The indicator represents an estimation of the year in which the paper would be published. Therefore, we designated the feature as “intrinsic publication year (IPY)”. Actually, IPY is defined as shown below.

$$\begin{array}{ccc} IPY(i) & = & w^{T}*v\left(i\right)+\epsilon \end{array}$$ (3)

$$\begin{array}{ccc} w,\epsilon & = & \underset{w,\epsilon }{\text{arg}\;\text{min}}\sum_{i\in \{Y\left(i\right)>=C_{y}\}}{\left(Y\left(i\right)-w^{T}*v\left(i\right)-\epsilon \right)}^{2} \end{array}$$ (4)

#### Step 1: Calculate the growth direction

We first estimate the growth direction using linear multiple regression analysis with a latent vector **v**(*i*) of each paper *i* as an explanatory variable and with publication time *Y*(*i*) as a target variable (shown in Eq (4)). Linear multiple regression is used because of the experimentally proved result that academic fields grow linearly in each dimension of latent space. Moreover, in the early stages of research within an academic field, there are insufficient data to ascertain a trend. Therefore, we use papers published after a certain cutoff year *C*_*t*_. After linear regression, we obtain parameter **w** and error *ϵ*.

#### Step 2: Calculate the intrinsic publication year (IPY)

Subsequently, we calculate each paper’s *IPY*(*i*) by fitting the latent representation vector **v**(*i*) to the acquired linear regression model, as shown in Eq (3). Here, IPY represents the position of a paper in the growth direction.

### 5.3 Validity of the proposed model

To confirm the validity of making linear growth model of a citation network in latent space obtained using LINE, we investigate the linear growth of a Barabási Albert (BA) network [33], which is a simple abstraction of a citation network, in latent space obtained by LINE. Consequently, the BA network grows almost linearly in latent space, especially using LINE 1st (see S1 File). Therefore, we use LINE for calculating the latent representation of citation networks.

### 5.4 Datasets

To ensure the general applicability of the results, we used a dataset of the American Physical Society (APS) and datasets of some domains in Web of Science provided by Thomson Reuters. The list of datasets is shown in Table 3. The APS dataset comprises about 597,000 papers related to physics and about 7 million citations between papers. These data include papers of the American Physical Society, the most prominent publisher in physics. The data from Web of Science are vast, including more than 100 million papers. Therefore, Web of Science data was narrowed down by queries created by specialists in their respective fields. These queries are the same as those used for earlier studies [9, 30].

**Table 3:** Datasets: Citation networks.

Name	Database	Query	Year	#Nodes	#Edges
APS	APS	Whole dataset	2016	596,786	7,074,825
Nanocarbon	WoS	(carbon and (nano* OR micro*)] or fullerene or Buckminsterfullerene or Buckminster-fullerene or C60 or C-60 or graphene or (filament* and carbon)	2016	482,596	6,005,416
Solar cells	WoS	Solar cell* or photovoltaics	2016	151,666	1,799,508

## Supporting information

S1 File Linear growth of BA model in latent space obtained by LINE. (DOC)
