# Supplementary material for: Detecting trends in academic research from a citation network using network representation learning
Source: PLoS One. 2018 May 21;13(5):e0197260. doi: 10.1371/journal.pone.0197260 (PMC5962067; doi:10.1371/journal.pone.0197260)
Supplement: S1 File — (DOC) [file pone.0197260.s001.doc]

**S1 File. Linear growth of BA model in latent space obtained by LINE**

For confirming the affinity of LINE to make a linear growth model of the network in latent space obtained by it, we investigate the *R*^2^ score of linear regression to predict the log values of the node number in order of the created time from each node’s representation vector. The log value of the node number corresponds to a published year in a real dataset because the number of published reports increases exponentially in a growing academic field.

The parameters are the following.

Parameters: BA model

*M* (number of nodes) = 1000, 4000, 16000

*N* (number of edges to attach from a new node to existing nodes) = 8, 16, 32

Parameters: LINE

Method: LINE 1st, LINE 2nd

Negative sampling rate: 8

Number of iterations: 1000

Results:

*R*^2^ scores of the fitting accuracy of the linear regression model in various conditions are almost 0.6–0.9 using LINE 1st and 0.3–0.8 in LINE 2nd. This high fitting using LINE 1st is supporting evidence to use LINE 1st for modeling the linear growth of a citation network which has a similar structure to that of the BA network. In the condition of low parameter *M*, the model does not perform very well, especially for large *N*, which indicates the need to input a dense network to the model. The reason for a low *R*^2^ score using LINE 2nd is regarded as attributable to the proximity function of LINE 2nd in which the neighbor nodes are not always located close to one another. However, the accuracy is presumed to be sufficiently large to model the linear growth of the network in latent space obtained by LINE 2nd.
